# Towards population-level conservation in the critically endangered Antarctic blue whale: the number and distribution of their populations

**DOI:** 10.1038/srep22291

**Published:** 2016-03-08

**Authors:** Catherine R. M. Attard, Luciano B. Beheregaray, Luciana M. Möller

**Affiliations:** 1 School of Biological Sciences, Flinders University, GPO Box 2100, Adelaide, SA 5001, Australia; 2 Department of Biological Sciences, Macquarie University, Sydney, NSW 2109, Australia

## Abstract

Population-level conservation is required to prevent biodiversity loss within a species, but it first necessitates determining the number and distribution of populations. Many whale populations are still depleted due to 20th century whaling. Whales are one of the most logistically difficult and expensive animals to study because of their mobility, pelagic lifestyle and often remote habitat. We tackle the question of population structure in the Antarctic blue whale (*Balaenoptera musculus intermedia*) – a critically endangered subspecies and the largest extant animal – by capitalizing on the largest genetic dataset to date for Antarctic blue whales. We found evidence of three populations that are sympatric in the Antarctic feeding grounds and likely occupy separate breeding grounds. Our study adds to knowledge of population structure in the Antarctic blue whale. Future research should invest in locating the breeding grounds and migratory routes of Antarctic blue whales through satellite telemetry to confirm their population structure and allow population-level conservation.

Population-level conservation is increasingly being recognized as a requirement for preventing biodiversity loss^1^. Ecological and evolutionary processes operate on a population level, therefore populations need to be conserved for ecosystems to continue to function and for organisms to adapt to today’s rapidly changing environment^2^,^3^,^4^. Knowledge of population structure – such as the number of populations, their distribution and their degree of interbreeding – is needed to define population-based units for management and conservation purposes.

Baleen whales were killed in the hundreds of thousands during 20th century whaling. It has long been recognized that their conservation should operate on a population level^5^. Understanding their population structure is complicated because of their pelagic and highly mobile lifestyle^6^. They typically feed at higher latitudes during summer and migrate to breed at lower latitudes during winter. The population structure possibilities span from each population having a separate non-breeding ground or grounds, to sharing of a non-breeding ground or grounds between different populations. Populations probably remain distinct in the latter case due to individuals returning to where they were born in order to breed. An additional challenge in unravelling the population structure of baleen whales for those feeding off Antarctica is their circumpolar distribution because it inhibits forming *a priori* boundaries of populations for analysis. Also, Antarctic baleen whales are highly expensive and logistically difficult to study. This is because of the remote and extreme weather conditions of the Antarctic, the high level of expertise required for expeditions, and the effort it takes to locate rare animals in an area encompassing millions of square kilometres.

Research methods have and continue to be developed to mitigate the intrinsic difficulties in studying the population structure of baleen whales. A method used during whaling was Discovery marking, where a whale was marked with a uniquely numbered Discovery tag and the tag recovered later when the whale was killed^7^. This provided two locations during the months and from the geographic areas of whaling, which was typically the austral summer and around Antarctica for baleen whales feeding in the Antarctic^8^. Additional technology emerged that allowed more than two locations of an individual: photo-identification by natural markings^9^,^10^ and satellite tagging^11^,^12^. Satellite tagging allows remote tracking of movements and continues to be optimized to increase tag longevity^12^,^13^. Acoustic recordings of vocalizations from whales have also been used to putatively identify populations based on geographically distinct calls and, when these calls occur repetitively, songs^14^,^15^,^16^ that are only known in males^17^,^18^,^19^,^20^. However, a lack of acoustic differences does not necessarily equate to a lack of population structure, and therefore requires confirmation using genetic approaches^21^. More cost-effective, informative, and high-throughput molecular markers continue to be developed, with microsatellites, mtDNA, and SNPs widely used today^22^. Genetic samples can be collected by remote biopsy sampling of live whales, and populations can be identified based on genetic differentiation between groups of individuals in accordance with population genetic theory^23^.

The largest animal is a species of baleen whale, the blue whale (*Balaenoptera musculus*), and the largest subspecies of blue whale is the Antarctic blue whale (*B. m. intermedia*)^24^,^25^. Antarctic blue whales reduced in abundance from 239,000 before hunting commenced in the 1904/05 austral summer season to a low of 360 when they were last hunted in the 1972/73 season^26^. The most recent abundance estimate was 2,280 from surveys conducted between the 1992/93 and 2003/04 austral summer^27^. This is only about 1% of pre-exploitation abundance. The subspecies is classified as Critically Endangered in the International Union for Conservation of Nature (IUCN) Red List of Threatened Species.

Under the International Whaling Commission (IWC), Antarctic blue whales are currently protected from commercial whaling and have not been killed under Special Permit (scientific) whaling. However, other anthropogenic activities may impede their recovery in numbers. Climate change may have consequences throughout the distribution of all populations^28^. At the feeding grounds in the Antarctic, potentially threatening anthropogenic activities include research operations, tourism, and the krill fishery^29^. At the breeding grounds and during migration, threats include but are not limited to seismic exploration for offshore oil and gas^30^, shipping noise^31^, and entanglement in fishing gear^32^. If Antarctic blue whales comprise of different populations, they would presumably inhabit different lower latitude areas during breeding and migration, so each population would be under a different array of anthropogenic threats outside the feeding season. Differences in threats between populations makes population-level conservation especially important to allow the persistence of each population.

The population structure of Antarctic blue whales can currently only be assessed where they feed, in the Antarctic, due to very limited knowledge of the locations of their breeding grounds. Movement of Antarctic blue whales studied using Discovery marks, photo-identification, and satellite tagging show large-scale movements as well as a degree of site fidelity to areas off Antarctica^33^,^34^,^35^,^36^,^37^,^38^. There are no known differences in song types within Antarctic blue whales^39^, which suggests that any populations within Antarctic blue whales are not acoustically distinct. Multiple populations of Antarctic blue whales are suspected based on analyses of genetic samples from the Antarctic that showed significant genetic differences at fixation indices between some IWC management Areas^40^. Defining management Areas was discussed since the 1930s and the Areas finally implemented from the 1974/75 season onwards by the IWC to aid in management of Southern Hemisphere baleen whales^41^. Each Area encompasses 50° to 70° longitude and stretches in latitude from the South Pole to the equator. Fixation index analyses require *a priori* putative populations, which are often geographic-based, and those that have been used (the IWC management Areas) as well as any other potential *a priori* geographic groupings are not necessarily biologically relevant given the continuous distribution of blue whales around the Antarctic. There has been no evidence of multiple populations based on Bayesian clustering assignment analyses^40^,^42^,^43^, which are a standard genetic method to assess population structure that does not require *a priori* geographic groupings. The lack of evidence could be due to insufficient sample sizes (*n* = 47^42^), insufficient number of markers (seven microsatellite loci^40^,^42^), or inclusion of samples from the pygmy blue whale subspecies (*B. m. brevicauda*)^42^,^43^.

Genetic samples of Antarctic blue whales have been collected since 1990 through highly costly and logistically difficult boat-based surveys conducted through the IWC. Genetic data collection from these samples should be maximized to provide high quantity and quality data that will inform conservation of this critically endangered animal. Here we examine the poorly defined population structure of Antarctic blue whales at their feeding grounds using the largest genetic dataset to date – 142 individuals of the Antarctic subspecies and information from 20 microsatellite markers and the mtDNA control region. We report novel findings that should be used to direct future research on the population structure of Antarctic blue whales.

## Results

**Figure 1:**
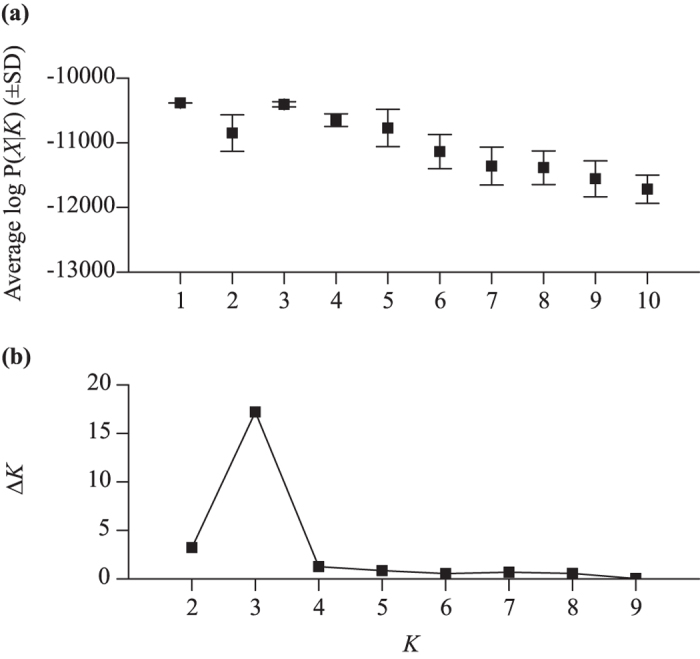
Inference of the number of genetic clusters detected by STRUCTURE for Antarctic blue whales using microsatellites. (**a**) Average estimate (±standard deviation) of the log of the probability of the data for each tested value of *K*, which was used to calculate (**b**) Δ*K* for tested values of *K*.

**Figure 2:**
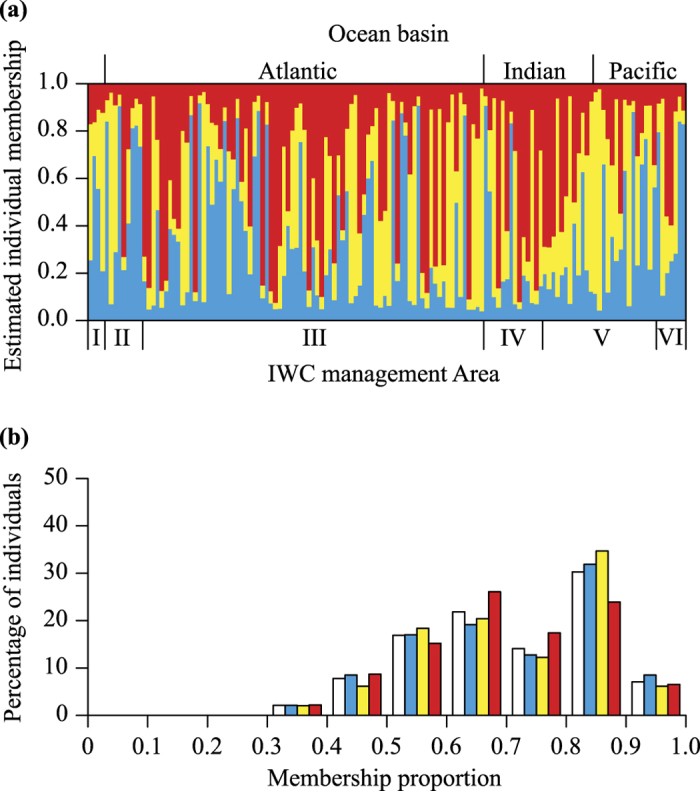
Clustering results of the STRUCTURE analysis for Antarctic blue whales using microsatellites when *K* is set to 3 and as summarized using CLUMPAK. Population 1, blue or medium grey in greyscale; population 2, yellow or light grey in greyscale; population 3, red or dark grey in greyscale. (**a**) Estimated membership of each individual to each cluster. Each individual is represented by a column. Individuals are ordered according to the longitude at which they were sampled, starting from the western border of IWC management Area I. Individuals sampled at the same longitude were ordered from the oldest to the newest sample. The colouring of each column represents the proportion of estimated membership of each individual to each population. (**b**) Percentage distribution histogram of the highest membership proportion of Antarctic blue whales (white) and blue whales from each population.

**Figure 3:**
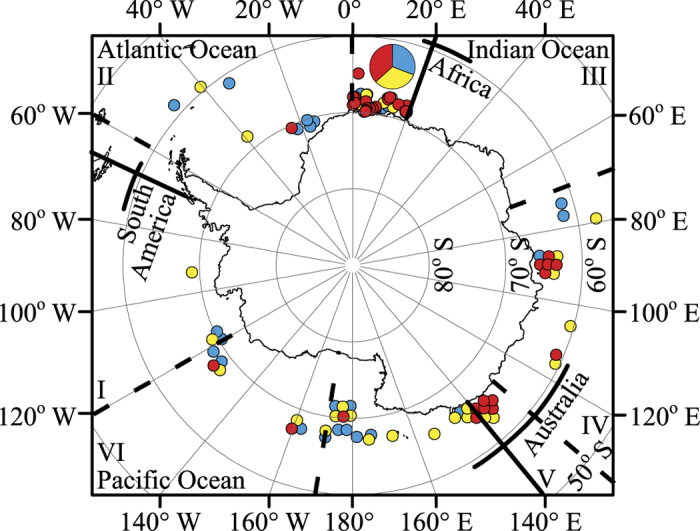
Polar map showing the biopsy sampling locations of individual blue whales off Antarctica. Each individual is represented by a circle, which is shaded according to its highest cluster membership as estimated in STRUCTURE using microsatellites (population 1, blue or medium grey in greyscale; population 2, yellow or light grey in greyscale; population 3, red or dark grey in greyscale). Only the first recorded location is shown for individuals sampled more than once. The map position of individuals sampled from the same or similar locations has been slightly altered so that all individuals are visible, with the exception of individuals located from 0° to 20°E due to extensive sampling from this area (*n* = 81). A pie chart shows the proportion of individuals that belong to each STRUCTURE cluster in the area from 0° to 20°E. Borders of IWC management Areas I to VI (dashed, black latitudinal lines) and ocean basins (solid, black latitudinal lines; borders according to International Hydrographic Organization definitions^110^) are shown. The longitudes of the southern coasts of South America, Africa and Australia are indicated (solid, black longitudinal lines). The map was created using ARCGIS 10.0 (Esri).

The Bayesian analysis of STRUCTURE inferred three genetic clusters that occur in sympatry off Antarctica. First, the likelihood in STRUCTURE across different values of *K* showed a peak at a *K* of three (−10406.48 (SD 39.99)) and one (−10385.12 (SD 0.56)) (Fig. 1). Second, the Δ*K* method estimated three genetic clusters (Δ*K*_MAX_ = 17.20; Fig. 1). Third, there was unequal (i.e. not approximately one third) estimated membership of each individual to each genetic cluster at a *K* of three, with some individuals strongly classified into a cluster (Fig. 2). Fourth, there was multiple evidence that clusters were not spurious. There was uneven geographic distribution of clusters; in the Pacific Ocean basin sector of the Antarctic there was evidence of a lower proportion of cluster 3 as only three of the 26 individuals there were identified as belonging to that cluster (Fig. 3). Additionally, these individuals had low maximum memberships of 0.547 to 0.597 inclusive, which means there may be no ‘pure’ individuals from this cluster in the Pacific Ocean basin sector of the Antarctic. All ten simulated panmictic populations had evidence of only one genetic cluster in STRUCTURE based on the likelihood peaking at a *K* of one (mean −10385.13 to −10385.40) and equal membership of individuals to each genetic cluster when *K* was set to more than one.

For the meaningful *K* value of three, CLUMPAK identified one major clustering solution (21 of 30 replicate runs; Fig. 2) and two minor clustering solutions (each 3 of 30 replicate runs). The relatively low maximum memberships of most individuals for the two minor clustering solutions indicate these clustering solutions are biologically infeasible. They are likely due to difficulty in searching the space of possible membership values.

Genetic variation was similar between each inferred cluster detected by STRUCTURE. This was regardless of whether variation was based on microsatellites or the mtDNA control region (Table 1). There was significant and similar genetic differentiation based on fixation indices between the clusters (microsatellite *F*_ST_ = 0.020–0.024; mtDNA *F*_ST_ = 0.016–0.029) (Table 2). There was also significant genetic differentiation between some IWC management Area pairwise comparisons (Table 2).

## Discussion

Our study helps refine knowledge of population structure in the Antarctic blue whale, *B. m. intermedia*. A Bayesian clustering assignment method based on microsatellite DNA provided evidence that three genetically differentiated populations occur sympatrically off Antarctica during the austral summer feeding season. For simplicity, these are henceforth referred to as populations. The levels of differentiation were low and similar between the populations, and consistent between analyses of microsatellites and the mtDNA control region. Genetic variation based on microsatellites and the mtDNA control region was similar in the three populations.

Previous studies were unable to detect the multiple, sympatric populations of Antarctic blue whales, even though they also used the same standard Bayesian clustering method^40^,^42^,^43^. This is because of either smaller sample sizes, smaller number of markers, not specifically investigating population structure within the Antarctic subspecies, or a combination of these. Fixation indices, which require *a priori* putative populations, have previously^40^ and in the current study detected genetic differentiation between some IWC management Areas. Given the results of the current study for analyses that do not require *a priori* groupings, this genetic differentiation between Areas could be due to different proportions of the populations in different management Areas.

The only longitudinal range where the proportion of different populations can be accurately assessed is between 0° and 20°E since 57% of samples were from this area. Here, there is an almost equal ratio of individuals from each population (number of samples from population 1 = 25, population 2 = 26, population 3 = 30). In contrast, in the Pacific Ocean basin sector there is perhaps greater proportions of populations 1 and 2 compared with population 3 as only three of the 26 individuals there were identified as belonging to population 3, and these had low memberships (estimated in STRUCTURE) to population 3 of 0.547 to 0.597 inclusive. These individuals and others with relatively low membership to their assigned population may have admixed ancestry to multiple populations^43^; in other words, they may have immediate ancestors (e.g. parents, grandparents) from different populations. An alternative explanation for low membership is insufficient power for accurate assignment given the low levels of population differentiation.

The movement data available for Antarctic blue whales corroborate the current study’s findings. They show large-scale movements off Antarctica – including movements that cross IWC management Areas and ocean basins – as well as small-scale movements. The data are from a wide range of methodologies and are across a wide time span: Discovery marks deployed from the 1934/35 to 1962/63 season and recaptured until the 1966/67 season^33^, photo-identifications from the 1987/88 to 2014/15 season^34^,^35^,^36^,^37^, Olson pers. comm. and satellite tagging and tracking within the 2012/13 season^38^ (Table 3). Longitudinal movements around the Antarctic require less travelling due to the high latitudes than the same amount of longitudinal movement at low latitudes, allowing the blue whales to move relatively easily between longitudes when in the Antarctic. The geographic distance was typically closer for intra-seasonal recaptures compared with inter-seasonal recaptures, though inter-seasonal recaptures still included small-scale movements^33^,^35^. The small-scale movements indicate site fidelity and the potential for different proportions of populations in different areas, and the large-scale movements indicate the potential for sympatry of populations.

The individual whales likely move depending on the densities of their prey due to their high energetic requirements as the largest extant animal^44^. Blue whales are specialist predators that feed on krill (order Euphausiacea). The Antarctic has high biological productivity, including Antarctic krill (*Euphausia superba*)^45^, due to the Antarctic Circumpolar Current generating upwelling and circumpolar fronts^46^. In the summer feeding season the Antarctic blue whales are generally south of the Antarctic Polar Front (also known as the Antarctic Convergence)^33^, a region associated with particularly high biological productivity^47^ and where blue whales feed on Antarctic krill^48^. The distribution and density of Antarctic krill changes within and between seasons depending on environmental conditions^49^, requiring marine predators, such as blue whales, to move to find sufficient amounts of their prey e.g.^50^,^51^,^52^. Such dependence is particularly important to consider given evidence of changes in Antarctic krill demographics due to recent climate change^53^,^54^. The dependence of blue whales on high concentrations of krill may also occur outside their feeding grounds^33^,^55^,^56^, unlike traditional thinking that baleen whales fast during migration and at breeding grounds. This means the specific locations of Antarctic blue whale breeding grounds may be influenced by the location and abundance of krill during the austral winter.

The exact breeding ground locations of Antarctic blue whales are unknown. One possibility is that populations breed at different ocean basins. This means that populations would be physically separated by the continental land masses of South America, Africa and Australia during the austral winter breeding season. It has long been suggested that at least one Antarctic population breeds in each of these physically separated regions^57^. Acoustic recordings of Antarctic blue whale calls in the austral winter indicate their breeding grounds include low latitudes of the Indian Ocean and eastern Pacific Ocean^58^,^59^. Their breeding grounds may also include low latitudes of the South Atlantic Ocean, with perhaps no recordings of their calls in that basin due to insufficient effort^39^. For example, the Atlantic Ocean region off south-west Africa has been suggested as a breeding location based on seasonality of Antarctic blue whale historical catches in south-west Africa^24^,^33^. However, since whaling there have been only two recorded blue whale sightings off that coast^33^. There is also a paucity of data on the population structure of other Antarctic baleen whales. There is evidence that populations of humpback whales (*Megaptera novaeangliae*) – arguably the most well-understood Antarctic baleen whale – have discrete distributions in the Antarctic, with overlap in the distribution of some populations^60^. They are thought to breed at lower latitudes segregated across the three ocean basins, but also with multiple populations breeding discretely in each ocean basin^60^. The Antarctic minke whale (*B. bonaerensis*) may also have a similar pattern of discrete population distributions in the Antarctic with some overlap^61^. Outside the Antarctic there is evidence of populations in sympatry at feeding grounds. The common minke whale (*B. acutorostrata*) appears to have complete sympatry on feeding grounds in the North Atlantic^62^.

It is also possible that at least one population of Antarctic blue whales is resident in the Antarctic throughout the year. A resident population may follow the ice edge as it expands northwards in the austral winter and recedes in the austral summer. This is suggested by year-round acoustic detections of the Antarctic subspecies off the western coast of the Western Antarctic Peninsula^63^,^64^ and off eastern Antarctica at 67°S, 70°E^64^. Antarctic blue whales have also been recorded year-round off the Crozet Islands^59^,^65^ based on acoustics, and off the sub-Antarctic island of South Georgia based on whaling catch data^33^. Blue whales of other subspecies have also been recorded at some localities year-round^59^,^65^,^66^,^67^,^68^, and other baleen whale species have been recorded in the Antarctic^69^,^70^,^71^ and other locations^72^,^73^,^74^,^75^ year-round. Some of these are likely to be resident populations^72^,^73^.

Another explanation for year-round presence at a feeding ground is overlapping timing of departures and arrivals. This overlap could be mediated by temporal segregation of migration based on age, sex, reproductive state, or migratory destination, as is thought for humpback whales^76^,^77^. Alternatively, a proportion of a population or populations may remain at the feeding grounds year-round. These could be individuals that are sexually or physically immature, or as also suggested for humpback whales^78^, mature females that are currently not breeding. Sexual maturity in blue whales is only reached at 10 years, and physical maturity afterwards^79^,^80^,^81^. Female blue whales are not thought to breed each season as they have a two to three year inter-calf interval, gestation lasting at least 10 months, weaning lasting seven months, and simultaneous pregnancy and lactation is rare^80^,^81^. However, the vocalizations that are geographically distinct in blue whales and appear as songs are likely only produced by males^17^,^18^, and the distinct Antarctic blue whale vocalization was used to report the year-round presence of blue whales in the Antarctic^63^,^64^. This means males are present year-round, with or without females.

It is possible that whaling has influenced the population structure detected here. Exploitation can lead to the formation or loss of populations, or for the level of genetic differentiation between populations to increase or decrease^82^. This can be due to increased genetic drift and associated loss of variation and increased genetic differentiation, and changes in the degree of gene flow between populations. Ideally, a sufficient number of pre-whaling samples from the same locations as the current samples should be used to determine if the population structure of blue whales has changed due to human impacts^83^, but such samples are not available in the present study. However, the most likely pre-whaling scenarios of blue whale populations can be inferred based on non-genetic data and the biology of this species. It is likely that there were multiple populations of Antarctic blue whales before whaling. While there are different possibilities regarding the breeding ground locations of Antarctic blue whales, acoustic and whaling catch data indicate that they include different ocean basins^24^,^33^,^58^,^59^. This would promote the formation of different populations, as seen in humpback whales^60^, because land barriers between ocean basins would hinder movement between breeding grounds. Philopatry to basins may be mediated by maternally-directed cultural learning of migratory routes and destinations, which makes population-level conservation particularly imperative as extirpation of a population could result in no re-colonization of the associated breeding ground^84^. In addition, it is likely that Antarctic blue whale populations were sympatric in the Antarctic before whaling. This is because there are no obvious differences in the pattern of blue whale movements in the Antarctic according to Discovery mark^33^ and photo-identification^34^,^35^,^36^,^37^ datasets, which together span from the 1934/35 to 2014/15 season. The non-genetic data therefore indicate that different populations likely existed within Antarctic blue whales before whaling, and these were sympatric in the Antarctic.

The current level of gene flow between populations may be greater than that prior to whaling, which could account for the low level of genetic differentiation and evidence of admixture found in the current study. Increased gene flow may occur because individuals need to travel further afield to find mates given that the number of Antarctic blue whales reduced from 239,000 to 360 individuals due to whaling^26^. Indeed, hybridization between the Antarctic blue whale and pygmy blue whale subspecies may have begun occurring or increased in occurrence within the last four decades due to whaling or climate change^43^. Although, there may have always been high levels of gene flow between the Antarctic populations. The low differentiation can otherwise be explained by the Antarctic populations being founded relatively recently (but still prior to whaling) on an evolutionary timescale, which is unlikely given evidence that Antarctic blue whales are the ancestral subspecies of blue whales in the Southern Hemisphere^85^. It is also unlikely that whaling has caused an increase in population differentiation through genetic drift because increased genetic drift has not yet existed for long enough to have a major effect; blue whales are long-lived with a generation time of 31 years^86^, and Antarctic blue whales were hunted from the 1904/05 to 1972/73 season, so the populations have only had a reduced size for about three to four generations.

Locating the breeding grounds of blue whales feeding off Antarctica is needed to confirm their current population structure. The biopsy samples used in the current study and the raw data for much of the discussed post-whaling Antarctic blue whale research, including photo-identifications^34^,^35^,^36^,^37^, abundance estimates^26^,^27^, past genetic research^40^,^42^,^43^, and satellite tagging^38^, were collected through international, collaborative vessel surveys in the Antarctic. The breeding grounds of Antarctic blue whales would ideally be located through continuing collaborative efforts to satellite tag Antarctic blue whales while they are feeding and tracking their subsequent movements^38^. Despite current longevity issues^12^, there has been much success in using tags to determine movements and migratory destinations^87^,^88^, and to assess inter-year and individual variation when enough tags are deployed^55^. In addition, tags continue to be improved to increase their longevity^12^,^13^. Subsequent genetic samples from breeding grounds would allow confirmatory genetic structure analyses with biologically reasonable *a priori* groupings and a baseline for comparative genetic analyses of samples collected off Antarctica. Monitoring each population’s abundance would then need to be performed at their breeding grounds. Monitoring is needed because the populations may differ in pre- and post-whaling abundance and recovery trends, especially because they occupy different migratory routes and breeding grounds with likely different carrying capacities and anthropogenic threats.

## Methods

### Data collection

Genetic samples from the 142 Antarctic blue whale individuals used in this study were collected off Antarctica using non-lethal biopsy darts. These samples exclude resamples, migrant pygmy blue whales, and subspecies hybrids found off Antarctica by Attard *et al*.^43^. The samples were collected from 1990 to 2009 in December to February during the International Decade of Cetacean Research (IDCR) and Southern Ocean Whale and Ecosystem Research (SOWER) cruises. This was approved by and conducted in accordance with the regulations of the IWC. Samples were preserved in either 20% DMSO saturated with NaCl or 70–100% ethanol, and archived at Southwest Fisheries Science Center (SWFSC) in the USA. DNA was extracted at SWFSC using multiple methods, including a modified salting-out protocol^89^ and DNeasy^®^ Blood and Tissue Kit (Qiagen).

Genetic data were collected from the samples at 20 microsatellites and a 414 bp fragment of the mtDNA control region. The data used in the current study from the 20 microsatellites were obtained by Attard *et al*.^43^ (BM032^90^ was discarded in the previous study and current study due to evidence of null alleles). Data from the 414 bp fragment of the mtDNA control region was obtained by LeDuc *et al*.^42^ and in the current study. The current study obtained the mtDNA data using the methods of LeDuc *et al*.^42^ or Attard *et al*.^90^, with the alteration of using an exo-SAP (Fermentas) protocol to purify mtDNA PCR products and an ABI 3730xl Genetic Analyzer (Applied Biosystems) for sequencing.

Analyses confirming microsatellite data adhered to the assumptions of subsequent analyses were conducted for the Antarctic subspecies and each genetic cluster identified by Bayesian analyses. This included checking for homozygote excess, and genotyping errors from stuttering and short allele dominance, using MICROCHECKER 2.2.3^91^ (95% confidence intervals, 10,000 runs). Deviations from Hardy-Weinberg equilibrium were tested using ARLEQUIN 3.5.1.2^92^ (10,000 dememorizations, 100,000 Markov chain steps) and linkage disequilibrium between pairs of loci were tested using FSTAT 2.9.3.2^93^, with sequential Bonferroni corrections applied to significance values^94^. The only potential evidence of unsuitability of a microsatellite locus was evidence of homozygote excess for locus Dde09^95^ for the whole dataset, but this was likely due to a lack of panmixia.

### Population structure

Population structure was assessed using a standard strategy that is suitable for continuously distributed and potentially sympatric populations. The Bayesian clustering assignment method of STRUCTURE 2.3.4^96^ was implemented for microsatellite data with the admixture model of ancestry, the correlated allele frequency model^97^, and without using sampling locations as priors^98^ (burn-in of 100,000 iterations then runs of 10^6^). Thirty independent runs were conducted for each value of *K* from one to 10, where *K* is the number of inferred genetic clusters. Runs were summarized using CLUMPAK^99^ (Main Pipeline, default parameters) to obtain the estimated membership of each individual to each cluster. CLUMPAK accounts for differences in replicate runs caused by label switching (from the arbitrary labelling of clusters) and multimodality (from either difficulty in searching the space of possible membership values or real biological factors).

Multiple measures were used to infer the most meaningful number of populations (*K*) detected by STRUCTURE. First, the probability of the data for each tested value of *K* was taken into account, with the highest probabilities expected at meaningful values of *K*. Second, the Δ*K* method^100^ was implemented in STRUCTURE HARVESTER 0.6.93^101^. As this method is based on changes in the probability of successive values of *K*, it can only detect a true *K* that is greater than one. Third, the distribution of estimated fraction of membership to each cluster for each individual at a given *K* was taken into account. Approximately 1/*K* fractions are expected for each individual at *K* > 1 when there is no genetic structure. Fourth, spurious genetic clustering was considered a possibility if the geographic distribution of inferred genetic clusters was even or if STRUCTURE detected two or more clusters for any of ten simulated panmictic populations.

The panmictic populations were simulated and analyzed following Quintela *et al*.^102^. Ten panmictic populations were simulated by permuting alleles within each locus using package *poppr*^103^ in R 3.0.3^104^ (function *shufflepop*, permute alleles method). These were analyzed in STRUCTURE using the models, burn-in and run length implemented for the empirical dataset. Ten independent runs were conducted for each value of *K* from one to five. The most meaningful *K* was determined based on the probability of the data for each tested value of *K* and the estimated fraction of membership to each cluster for each individual at a given *K*. The Δ*K* method was not implemented as it can only detect a true *K* that is greater than one.

Pairwise genetic differentiation (*F*_ST_) was calculated based on microsatellites and the mtDNA control region using ARLEQUIN (significance assessed by 10,000 permutations) between the clusters, as well as between *a priori* geographic groupings of IWC management Areas. Genetic differentiation was calculated to allow comparisons with previous *a priori* genetic differentiation analyses of Antarctic blue whales^40^ rather than to elucidate the genetic structure of Antarctic blue whales. Exact tests of population differentiation were conducted for microsatellites using GENEPOP 4.3^105^,^106^ (10,000 dememorizations, 100 batches, 10,000 iterations per batch) and for the mtDNA control region using ARLEQUIN (100,000 Markov chain steps, 10,000 dememorizations).

### Genetic variation

Genetic variation was determined for the Antarctic subspecies and each genetic cluster identified by Bayesian analyses. Variation based on microsatellites was determined by calculating the number of alleles, observed heterozygosity (H_O_) and unbiased expected heterozygosity (H_E_) using GENALEX 6.501^107^,^108^, and allelic richness using FSTAT^93^. Genetic variation based on the mtDNA control region was determined by calculating the haplotype diversity (*h*) using ARLEQUIN, and haplotype richness using CONTRIB 1.02^109^.

## Additional Information

**How to cite this article**: Attard, C. R. M. *et al*. Towards population-level conservation in the critically endangered Antarctic blue whale: the number and distribution of their populations. *Sci. Rep.* **6**, 22291; doi: 10.1038/srep22291 (2016).

## Figures and Tables

**Table 1:** Genetic variation at 20 microsatellites and the mtDNA control region of Antarctic blue whales and the populations identified using STRUCTURE.

	Microsatellites					mtDNA control region			
	*n*	NA	H_O_	H_E_	AR	*n*	NH	*h*	HR
AllAntarctica	142	11.65(5.46)	0.758(0.130)	0.763(0.133)	9.99	140	46	0.968(0.005)	26.58
Population 1	47	9.85(4.04)	0.764(0.148)	0.761(0.148)	9.82	46	23	0.958(0.013)	22.76
Population 2	49	9.05(3.72)	0.751(0.144)	0.748(0.130)	8.96	49	25	0.958(0.014)	23.97
Population 3	46	9.50(4.65)	0.760(0.122)	0.747(0.126)	9.50	45	20	0.938(0.017)	20.00

*n*, number of samples; NA, mean number of alleles; H_O_, mean observed heterozygosity; H_E_, mean unbiased expected heterozygosity; AR, mean allelic richness (based on the minimum sample size of 46 across populations and loci); NH, number of haplotypes; *h*, haplotype diversity; HR, haplotype richness (based on the minimum sample size across populations). Standard deviations are in parentheses.

**Table 2:**
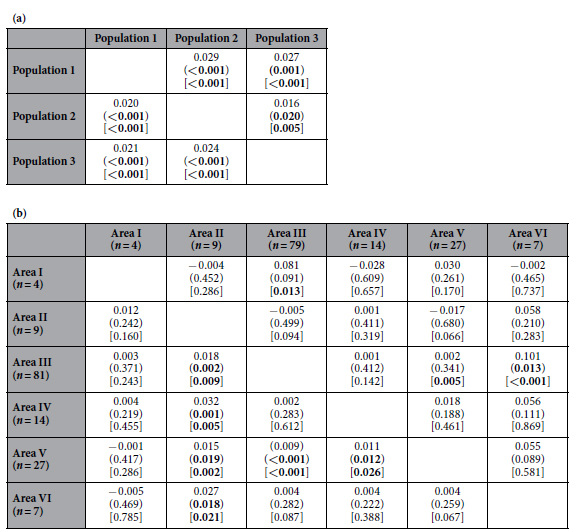
Genetic differentiation (*F*_ST_) of Antarctic blue whales between (a) the populations identified using STRUCTURE, and (b) the IWC management Areas.

Analyses were conducted based on microsatellites (below diagonal) and the mtDNA control region (above diagonal).

*P* values from permutation are in parentheses and from exact tests are in square brackets. Significant *P* values are in bold.

**Table 3:** Summary of movement data for Antarctic blue whales generated by previous studies through Discovery marking, photo-identification and satellite tagging.

	Discovery marks^33^	Photo-identifications^34^,^35^,^36^,^37^, Olson pers. comm.	Satellite tagging^33^
Austral summer seasons	1934/35–1966/67	1987/88–2014/15	2012/13
Number of individuals	2295	399	2
Number recaptured within a season	49	36	NA
Minimum movement within a season(degrees longitude; km)	0.10; 32	0.04; 3	16.12; 1433
Maximum movement within a season(degrees longitude; km)	76.45; 3516	24.69; 1172	75.06; 5300
Number recaptured between seasons	46	14	NA
Minimum movement between seasons(degrees longitude; km)	1.77; 122	0.47; 19	NA
Maximum movement between seasons(degrees longitude; km)	172.58; 6250	141.81; 6550	NA

The distances provided as km include latitudinal movements, and the movements within a season exclude those recorded on the same day. The “number of individuals” for Discovery marking is the number of mark deployments, however note that four individuals recaptured within a season, four recaptured between seasons, and one recaptured on the same day had two marks, and that one recaptured between seasons was morphologically identified as a pygmy blue whale^33^. The whale with the minimum movement detected by satellite tagging was also the whale with the maximum intra-seasonal photo-identification, with a photo-identification taken prior to tagging and on the day of tagging^36^. Two of the inter-seasonal photo-identification recaptures^35^ were also identified as recaptures using genetic methods^40^,^43^. One was a female identified in the current study as belonging to population 3 (estimated membership 0.474) and moved 131.70° longitude, the other was a male identified as belonging to population 1 (estimated membership 0.846) and moved 6.69° longitude. NA, not applicable.
